# Maximum Acceptable Tilt Angle for Point Autofocus Microscopy

**DOI:** 10.3390/s23249655

**Published:** 2023-12-06

**Authors:** Huixu Song, Qingwei Li, Zhaoyao Shi

**Affiliations:** Beijing Engineering Research Center of Precision Measurement Technology and Instruments, Beijing University of Technology (BJUT), Beijing 100124, China; liqwei@emails.bjut.edu.cn (Q.L.); shizhaoyao@bjut.edu.cn (Z.S.)

**Keywords:** point autofocus microscopy, small components, complex components, maximum acceptable tilt angle, reference sphere

## Abstract

The complete and accurate acquisition of geometric information forms the bedrock of maintaining high-end instrument performance and monitoring product quality. It is also a prerequisite for achieving the ‘precision’ and ‘intelligence’ that the manufacturing industry aspires to achieve. Industrial microscopes, known for their high accuracy and resolution, have become invaluable tools in the precision measurement of small components. However, these industrial microscopes often struggle to demonstrate their advantages when dealing with complex shapes or large tilt angles. This paper introduces a ray-tracing model for point autofocus microscopy, and it provides the quantified relationship formula between the maximum acceptable tilt angle and the beam offset accepted in point autofocus microscopy, then analyzing the maximum acceptable tilt angle of the objects being measured. This novel approach uses the geometric features of a high-precision reference sphere to simulate the tilt angle and displacement of the surface under investigation. The research findings show that the maximum acceptable tilt angles of a point autofocus microscope vary across different measured directions. Additionally, the extent to which the maximum acceptable tilt angles are affected by the distances of the beam offset also varies. Finally, the difference between the experiment results and the theoretical results is less than 0.5°.

## 1. Introduction

In the era of artificial intelligence, complex and tiny components have seen wide application, especially in strategic sectors [1,2,3] such as next-generation communications, service robots, automotive electronics, intelligent sensors, defence equipment and aerospace. Both the geometric accuracy and product quality of these components are crucial for ensuring stable and accurate system operations. Accurate evaluation of product characteristics and quality relies on obtaining sufficient information about their condition, which in turn depends on the continuous improvement of measurement theory and technological devices. Therefore, high-precision measurement methods are indispensable tools for guiding the high-precision processing of complex and small components, thereby improving product quality. Complex small components, despite their overall diminutive size, often possess geometric features with large depth-to-width or length-to-diameter ratios [4]. Traditional contact measurement methods struggle to effectively obtain comprehensive information about complex small surfaces [5]. Furthermore, for components with different sizes but equivalent accuracy levels, the tolerance requirements for smaller ones are stricter than those for larger ones [6,7]. This makes it challenging for non-contact measurement methods such as structured light and machine vision to meet the required measurement accuracy. In this context, point autofocus microscopy technology provides a better solution for measuring the geometric errors of complex small components [8,9].

Point autofocus microscopy can be used to measure various complex surfaces, especially for small machine elements, such as micro gears and micro splines. Similarly to other microscopic measurement systems, point autofocus microscopy has limitations, including a narrow measurement field of view and a small maximum acceptable tilt angle [10]. To effectively gather complete surface information from a complex and small component, multiple measurements and data stitching are typically required to keep the tested region within the microscopy’s maximum acceptable tilt angle [11,12]. However, this approach not only decreases the measurement efficiency but also amplifies potential errors [13,14,15]. Increasing the maximum acceptable tilt angle of the microscopic measurement systems can effectively reduce the need for data stitching and expand the measurement range. Simultaneously, it can improve the ability of point autofocus microscopy to measure the geometric information of complex surfaces. Nikolaev et al., 2016 conducted a study on the maximum acceptable tilt angle for a focus variation microscope, primarily focusing on the impact of different tilt angles of the measured surface on surface roughness measurements [16]. Thomas et al., 2021 investigated the maximum acceptable tilt angle for a coherence scanning interferometer by modeling a coherence scanning interferometer [17]. Gao et al., 2023 analyzed the maximum measurable tilt angle accepted by a confocal microscope under different numerical aperture (NA) objectives [18]. The maximum acceptable tilt angle of point autofocus microscopy is related to the offset distance and direction of the incident laser beam when the reflected laser beam from the workpiece remains within the receiving range of the objective [19]. Therefore, quantifying the relationship between the offset of the incident laser beam and the maximum acceptable tilt angle is crucial for expanding the measurement range of point autofocus microscopy.

This article expounds the principles of point autofocus microscopy and quantifies the relationship between the laser beam offset and the maximum acceptable tilt angle in the second part. In the third part, a maximum acceptable tilt angle measurement method based on a reference sphere and the parameters of several crucial components are given. In the fourth part, via theoretical calculations and experimental verification, we determine the maximum acceptable tilt angle of a point autofocus microscope with a sub-micrometre measurement accuracy. Finally, in the fifth part, the contents of this article are summarized and a reasonable point autofocus microscopy measurement scheme is given.

## 2. Basic Principle and Optical Model

### 2.1. Measurement Principle of Point Autofocus Microscopy

Point autofocus microscopy is a non-contact surface measuring tool that can automatically focus a collimated laser beam onto a target surface. This, in conjunction with the motion of a two-dimensional motion stage (2D motion stage), allows for dynamic scanning measurements of a surface [20]. Using an Olympus 100× objective (NA = 0.8), the focused spot can reach a minimum diameter of 1 μm. This data then enable a more in-depth analysis of the geometric accuracy of the measured surface.

**Figure 1:**
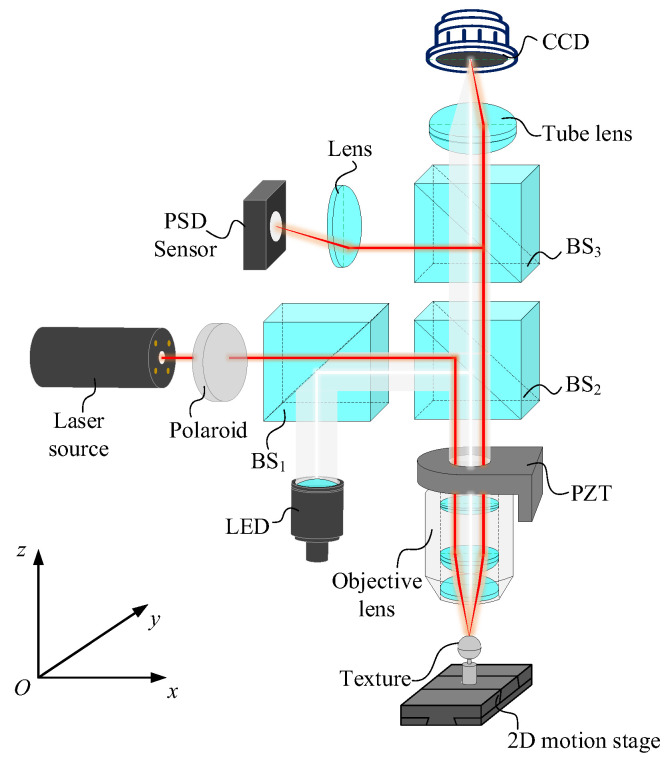
The schematic diagram of point autofocus microscopy.

Figure 1 illustrates the main structure of point autofocus microscopy, which comprises multiple components, including a laser source, LED source, beam splitter, objective, 2D motion stage, tube lens, focusing lens, PSD sensor, CCD camera (CCD, Charge Coupled Device), objective scanner (PZT, Piezoelectric Transducer) and motion control system. These components collectively form two systems: the laser measurement system and the white light imaging system. In the laser measurement system, the laser beam is reflected by the beam splitter and transmitted through the objective at a certain offset distance from the optical axis of the objective. The focused laser beam forms a micron-sized spot on the workpiece surface. After reflecting off the workpiece, the laser beam passes through the objective again and travels through the beam splitter as collimated light. It is then focused by the focusing lens onto the centre of the PSD sensor. When the 2D motion stage moves to a new position, the laser spot on the workpiece no longer aligns with the focal plane of the objective. Therefore, the laser beams passing through the objective, beam splitter and focusing lens deviate from their original positions, and the final laser beam is no longer focused on the centre of the PSD sensor. This shift in the focused position of the laser spot generates the corresponding photoelectric signals in the PSD sensor. These signals control the objective scanner, moving it in the direction of the optical axis with nanometre precision until the spot returns to the centre of the PSD sensor again. The distance that the objective scanner moves along the optical axis represents the difference in distance between the two measured positions on the workpiece towards the optical axis. This, combined with the motion of the 2D motion stage, facilitates the measurement of the 3D surface. However, the white light imaging system works entirely differently. Here, the broadband light emitted by the LED source is reflected by the beam splitter and transmitted through the objective without any offset. The white light reflected from the workpiece is projected onto the imaging plane of the CCD camera after transmission through the objective and the tube lens. When the surface of the workpiece aligns with the focal plane of the objective, the clearest image of the surface appears on the CCD imaging plane. In other words, when the workpiece surface aligns with the focal plane of the objective, the laser beam is focused on the centre of the PSD sensor, presenting the clearest image of the surface on the CCD camera.

### 2.2. The Relationship between Laser Beam Offset and Maximum Acceptable Tilt Angles

**Figure 2:**
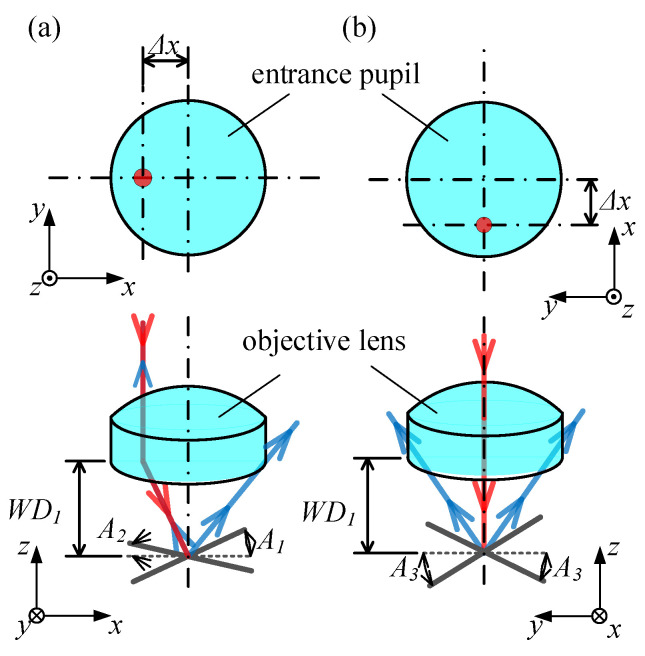
The relationship between laser beam offset and maximum acceptable tilt angles. (**a**) The measured surface tilts around the y-axis; (**b**) The measured surface tilts around the x-axis. The red arrow represents incident laser beam, and the blue arrow represents reflected laser beam.

The maximum acceptable tilt angles of the point autofocus microscope are dependent on the direction and distance of the laser beam offset. As depicted in Figure 2, when the laser beam is positioned in the negative x-axis direction with an offset of Δ*x*, *A*_1_ and *A*_2_ represent the maximum acceptable tilt angles for the clockwise and anti-clockwise rotation of the measured surface around the y-axis, respectively. Conversely, for both clockwise and anti-clockwise rotations of the surface measured around the x-axis, the maximum acceptable tilt angle is depicted at *A*_3_; however, *A*_3_ will change according to the direction and distance of the laser beam offset.

**Figure 3:**
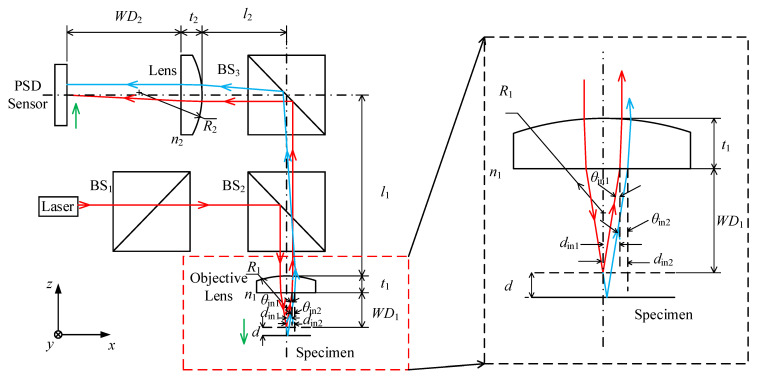
Optical model of point autofocus microscopy.

To quantify the maximum acceptable tilt angles under different directions and distances of the laser beam offset, an optical model of point autofocus microscopy is established, as shown in Figure 3. This model incorporates several key parameters. For the objective, these include a refractive index of *n*_1_, a working distance of *WD*_1_, a centre thickness of *t*_1_ and a curvature radius of *R*_1_. The focusing lens has a refractive index of *n*_2_, a working distance of *WD*_2_, a centre thickness of *t*_2_ and a curvature radius of *R*_2_. The distance between the objective and the centre of the BS_3_ is *l*_1_, while *l*_2_ represents the distance between the focusing lens and the centre of the BS_3_. In this model, the laser beam enters the optical system with the pose of [*d*_in_, *θ*_in_], and exits with the pose of [*d*_out_, *θ*_out_]. Here, *d*_in_ and *θ*_in_ represent the offset displacement and deflection angle of the laser beam reflected by the measured surface when it enters the objective with respect to the optical axis of the objective. Similarly, *d*_out_ and *θ*_out_ represent the offset displacement and deflection angle of the laser beam when it passes through the focusing lens and enters the PSD sensor relative to the optical axis of the focusing lens. Applying the principle of paraxial ray tracing (see (1)), it is possible to calculate the position of the spot on the PSD sensor [21,22].

(1) $$\begin{array}{c} d_{out}\;=\;\left[\begin{array}{cc} 1 & f_{2} \end{array}\right]\cdot \left[\begin{array}{cc} 1 & 0\\ 0 & n_{2} \end{array}\right]\cdot \left[\begin{array}{cc} 1 & t_{2}\\ 0 & 1 \end{array}\right]\cdot \left[\begin{array}{cc} 1 & 0\\ \frac{1-n_{2}}{n_{2}R_{2}} & \frac{1}{n_{2}} \end{array}\right]\cdot \left[\begin{array}{cc} 1 & l_{1}\\ 0 & 1 \end{array}\right]\\ \cdot \left[\begin{array}{cc} 1 & 0\\ \frac{n_{1}-1}{R_{1}} & n_{1} \end{array}\right]\cdot \left[\begin{array}{cc} 1 & t_{1}\\ 0 & 1 \end{array}\right]\cdot \left[\begin{array}{cc} 1 & 0\\ 0 & \frac{1}{n_{1}} \end{array}\right]\cdot \left[\begin{array}{c} d_{in}\\ \theta_{in} \end{array}\right] \end{array}$$

Figure 3 illustrates two propagation paths of the laser beam depending on the location of the measured surface. The solid red line represents the propagation path of the laser beam when the measured surface is located at the focal plane of the objective. Reflected by the measured surface, the laser beam enters the objective with the pose of [*d*_in1_, *θ*_in1_]. The reflected laser beam is focused onto the PSD sensor surface with the pose of [*d*_out1_, *θ*_out1_]. Alternatively, the solid blue line in Figure 3 represents the propagation path of the laser beam when the measured surface is moved a certain distance *d_in_* in the negative z-axis direction. Reflected by the measured surface, the laser beam enters the objective with the pose of [*d*_in2_, *θ*_in2_]. The reflected laser beam is focused onto the PSD sensor surface with the pose of [*d*_out2_, *θ*_out2_]. Formula (2) can be used to determine the position of the laser spot on the PSD sensor.

(2) $$d_{out2}\;=\;\left[\begin{array}{cc} A & B \end{array}\right]\cdot \left[\begin{array}{c} d_{in1}\\ \theta_{in1} \end{array}\right]\;=\;A\cdot d_{in2}+B\cdot \theta_{in2}$$

Here,

(3) $$\begin{array}{c} \left[\begin{array}{cc} A & B \end{array}\right]\;=\;\left[\begin{array}{cc} 1 & f_{2} \end{array}\right]\cdot \left[\begin{array}{cc} 1 & 0\\ 0 & n_{2} \end{array}\right]\cdot \left[\begin{array}{cc} 1 & t_{2}\\ 0 & 1 \end{array}\right]\cdot \left[\begin{array}{cc} 1 & 0\\ \frac{1-n_{2}}{n_{2}R_{2}} & \frac{1}{n_{2}} \end{array}\right]\cdot \left[\begin{array}{cc} 1 & l_{1}\\ 0 & 1 \end{array}\right]\\ \cdot \left[\begin{array}{cc} 1 & 0\\ \frac{n_{1}-1}{R_{1}} & n_{1} \end{array}\right]\cdot \left[\begin{array}{cc} 1 & t_{1}\\ 0 & 1 \end{array}\right]\cdot \left[\begin{array}{cc} 1 & 0\\ 0 & \frac{1}{n_{1}} \end{array}\right] \end{array}$$

According to the geometric characteristics of the optical model, *θ*_in1_ equals *θ*_in2_. Therefore, changes in the position of the laser spot on the PSD sensor can be determined using Formula (4).

(4) $$d_{out2}-d_{out1}\;=\;A\cdot \left(d_{in2}-d_{in1}\right)\;=\;A\cdot \Delta d_{in}$$

**Figure 4:**
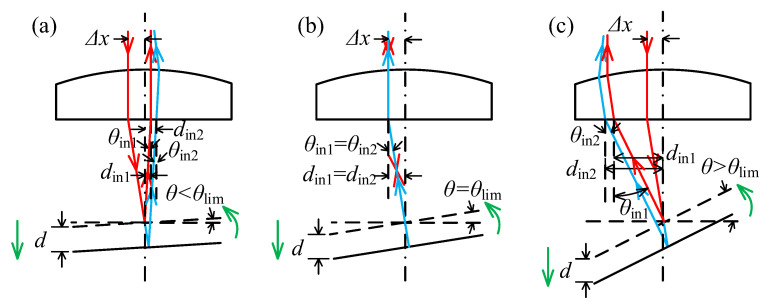
The relative position for the incident and reflected laser beam when the measured surface tilts anti-clockwise around the y-axis. (**a**) The tilt angle *θ* < *θ*_lim_; (**b**) The tilt angle *θ* = *θ*_lim_; (**c**) The tilt angle *θ* > *θ*_lim_. The red arrow represents the laser beam before the tilt of the measured surface, and the blue arrow represents the laser beam after the tilt of the measured surface.

Step 1: Figure 2a illustrates a scenario where the measured surface tilts anti-clockwise around the y-axis. As shown in Figure 4, *d*_in_ of the reflected laser beam increases as the surface moves away when the tilt angle *θ* < *θ*_lim_, resulting in Δ*d*_in_ > 0. When the tilt angle *θ* continues to increase to *θ*_lim_, the reflected laser beam aligns with the incident laser beam and *d_in_* remains constant irrespective of surface movements; hence, Δ*d*_in_ = 0. At this inclination angle, the PSD sensor fails to accurately determine the displacement and direction of the objective. However, if the tilt angle *θ* continues to increase, *d_in_* of the reflected laser beam decreases as the surface moves away, resulting in Δ*d*_in_ < 0. The position where the reflected laser beam aligns with the incident laser beam determines the maximum acceptable tilt angle *A*_1_, which can be calculated using Formula (5). In this formula, Δx represents the offset displacement of the incident laser beam on the objective.

(5) $$A_{1}\;=\;\arctan\frac{\Delta x}{WD_{1}}$$

**Figure 5:**
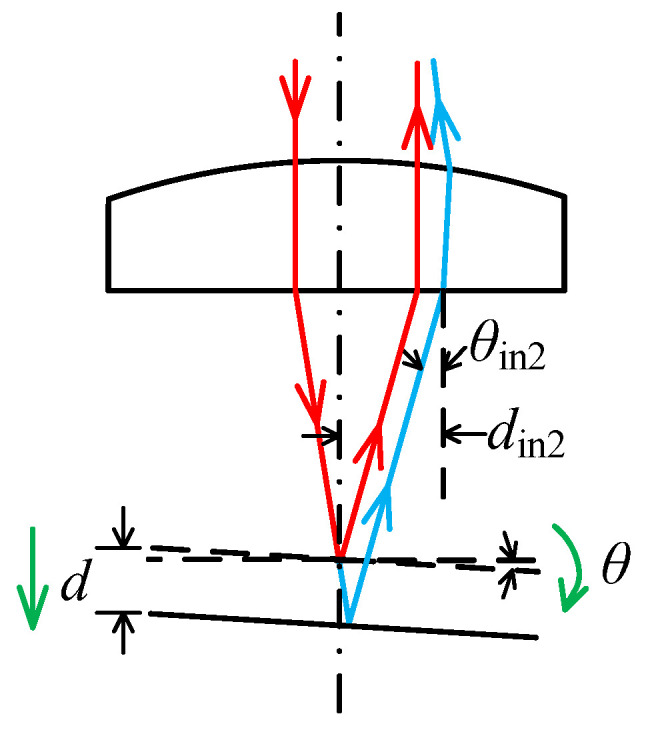
The relative position for the incident and reflected laser beam when the measured surface tilts clockwise around the y-axis. The red arrow represents the laser beam before the tilt of the measured surface, and the blue arrow represents the laser beam after the tilt of the measured surface.

Step 2: Figure 2a illustrates a scenario where the measured surface tilts clockwise around the y-axis. As shown in Figure 5, *d*_in_ of the reflected laser beam increases as the surface moves away and decreases as the surface moves closer. This indicates a consistent correlation between the motion direction of the laser spot on the PSD sensor and the motion direction of the measured surface. This is necessary to ensure that the laser beam reflected by the measured surface does not exceed the effective diameter range of the objective. The maximum acceptable tilt angle, *A*_2_, can be determined using Formula (6), where α represents the aperture angle of the objective.

(6) $$A_{2}\;=\;\frac{\frac{\alpha }{2}-\arctan\frac{\Delta x}{WD_{1}}}{2}$$

**Figure 6:**
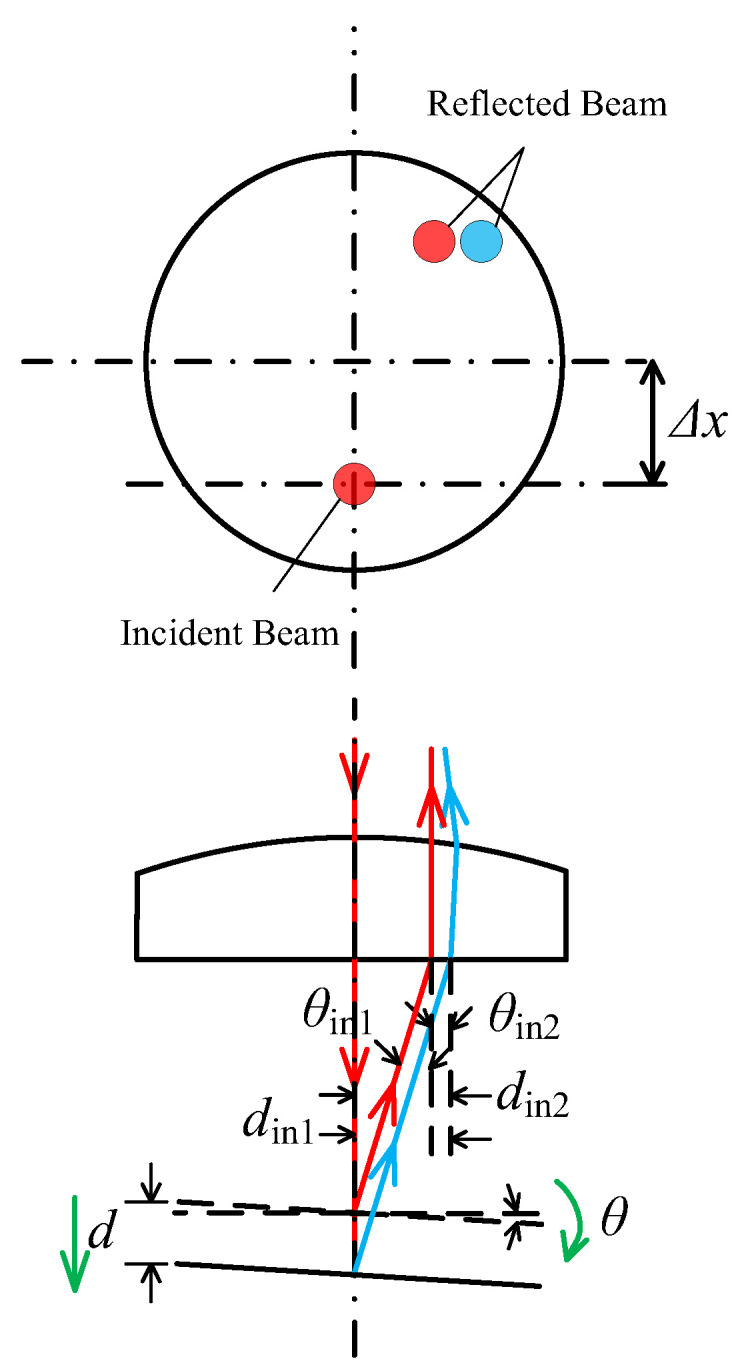
The relative position for the incident and reflected laser beam when measured surface tilts clockwise and anti-clockwise around the x-axis. The red arrow represents the laser beam before the tilt of the measured surface, and the blue arrow represents the laser beam after the tilt of the measured surface.

Step 3: Figure 2b illustrates a scenario where the measured surface tilts clockwise and anti-clockwise around the x-axis. As shown in Figure 6, |*d*_in_| of the reflected laser beam increases as the surface moves away and decreases as it moves closer. This demonstrates a distinct and fixed correlation between the motion direction of the laser spot on the PSD sensor and the motion direction of the measured surface. It is important to ensure that the laser beam reflected by the measured surface does not exceed the effective diameter range of the objective. The maximum acceptable tilt angle, *A*_3_, can be calculated using Formula (7). Here, *D* represents the effective diameter of the objective.

(7) $$A_{3}\;=\;\frac{\arctan\frac{\sqrt{{\left(\frac{D}{2}\right)}^{2}-{\left(\Delta x\right)}^{2}}}{WD_{1}}}{2}$$

## 3. Experimental Validation

### 3.1. Experimental Method

**Figure 7:**
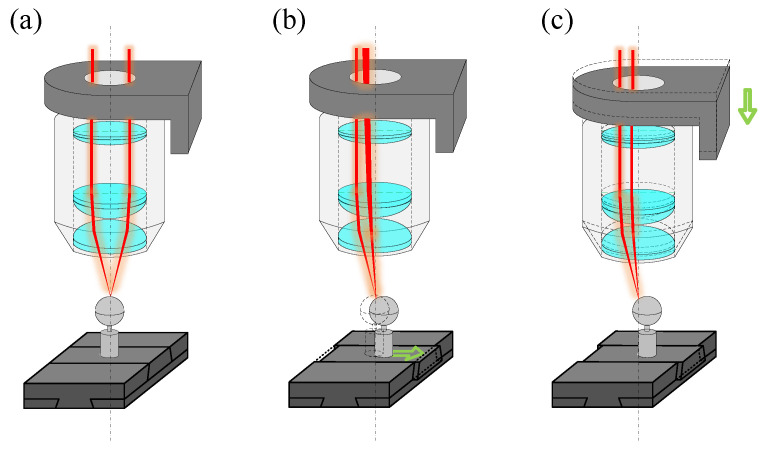
Schematic of the experimental process. (**a**) The initial status; (**b**) The motion of the reference sphere during measurement; (**c**) The motion process of the objective scanner during the measurement. The green arrow indicates the direction of component movement.

A maximum acceptable tilt angle is crucial in point autofocus microscopy, which measures its accuracy on curved or inclined surfaces. A reference sphere is used in the experiment (as shown in Figure 7) to simulate the displacement and tilt angle changes of the workpiece.

Figure 7a shows the initial status, where the centre of the reference sphere is located on the extension line of the optical axis of the objective. By adjusting the objective scanner, the apex of the reference sphere is located on the focal plane of the objective. Figure 7b shows the motion of the reference sphere during measurement. The reference sphere moves in the positive direction of the x-axis in incremental steps by controlling the 2D motion stage. This movement causes the measured point on the reference sphere to shift away from the objective, resulting in the deviation of the laser beam by the reference sphere. Consequently, it causes the spot on the PSD sensor to deviate from the centre. Figure 7c shows the motion process of the objective scanner during the measurement. The PSD sensor generates a deviation signal, processed and used to prompt the objective scanner to move in the negative direction of the z-axis. The objective approaches the reference sphere until the measured point on the sphere aligns with the focal plane of the objective. The distance moved by the objective scanner indicates the coordinate difference *z*_m_ (also known as the measuring value) between the two points measured on the reference sphere along the optical axis of the objective. Using the geometric parameters and horizontal motion distance of the reference sphere, we can accurately calculate the coordinate difference *z*_t_ (also known as theoretical value) between the two points measured along the optical axis of the objective (see (8)).

(8) $$z_{t}\;=\;R-\sqrt{R^{2}-x^{2}}$$

Simultaneously, we can also calculate the tilt angle at each measurement position of the reference sphere. *R* represents the radius of the reference sphere, and *x* signifies the displacement of the reference sphere along the x-axis. In the experiment, the 2D motion stage carries the reference sphere in 50 μm steps, moving in both the positive and negative directions of the x-axis, as well as the positive direction of the y-axis. A high-precision incremental-length gauge performs the precise displacement of the reference sphere. Given the sub-micrometre measurement accuracy of the system, the measurement error of the point autofocus microscopy is maintained within 1 μm. Ultimately, the tilt angles corresponding to the extreme error position are obtained and defined as the measured value *A*_m_ of the maximum acceptable tilt angles (see (9)).

(9) $$A_{m}\;=\;\arcsin\frac{x}{R}$$

### 3.2. Experimental System

**Figure 8:**
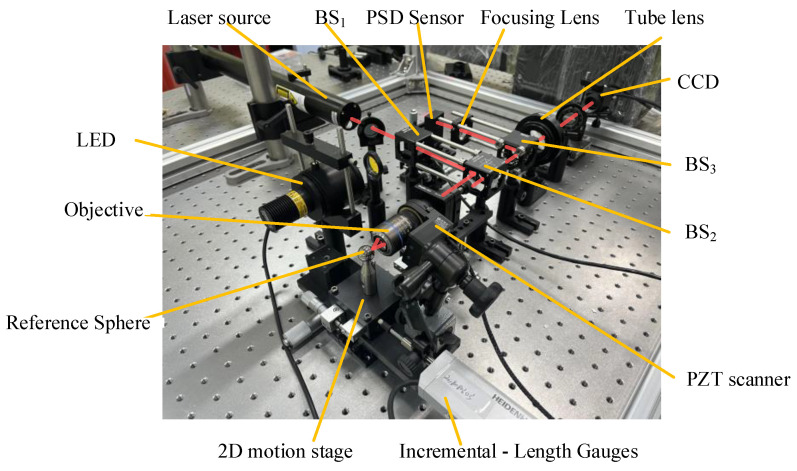
The protype of the experimental system.

This article presents an experimental method for quantifying the maximum acceptable tilt angles of point autofocus microscopy and constructs an experimental system, as shown in Figure 8. The accuracy of the entire experimental system relies on several crucial components. These include the objective, reference sphere, objective scanner, PSD sensor and high-precision incremental-length gauges. First, our objective is the LMPlanFL N 50 from Olympus, Tokyo, Japan, and its main technical parameters are listed in Table 1.

The reference sphere is an STL Precision Ball from Hexgon, Stockholm, Sweden, and its main technical parameters are listed in Table 2.

The objective scanner is P73.Z200S from COREMORROW, Harbin, China, and its main technical parameters are listed in Table 3.

The PSD sensor is the PDP90A from Thorlabs, Newton, NJ, USA, and its main technical parameters are listed in Table 4.

The high-precision incremental-length gauge is MT 2500 from Heidenhain, Traunreut, Germany, and its main technical parameters are listed in Table 5.

The experimental protype is shown in Figure 8.

## 4. Results and Discussion

Owing to the restriction of the objective’s entrance pupil and the diameter of the laser beam, the maximum offset of the laser beam is 4 mm. To verify the quantitative relationship between the offset and the maximum acceptable tilt angles, an experiment has been designed to measure the maximum acceptable tilt angles *A*_m1_, *A*_m2_ and *A*_m3_ at offsets of 1 mm, 2 mm, 3 mm and 4 mm, respectively. Each offset distance will undergo five repeated measurements to ensure data accuracy. The standard deviation *σ* of the measurement data and the expanded uncertainty *u* (k = 2) can be calculated using Formulas (10) and (11), respectively. *n* represents the number of repeated measurements, and *e*_i_ represents the measurement error for the *i*th (1, 2, 3, 4, 5) group at each measurement position. In order to achieve a sub-micrometre measurement accuracy, the expanded uncertainty *u* should be less than 1 μm.

(10) $$\sigma \;=\;\sqrt{\frac{\sum_{i=1}^{n}{\left(e_{i}-\bar{e}\right)}^{2}}{n-1}}$$

(11) $$u\;=\;k\cdot \frac{\sigma }{\sqrt{n}}$$

**Figure 9:**
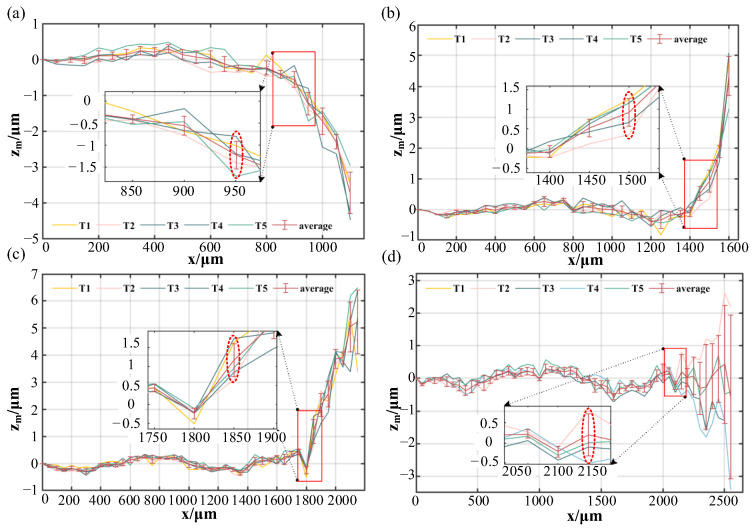
The measurement error when the measured surface tilts anti-clockwise around the y-axis. (**a**) The laser beam offset at 1 mm; (**b**) The laser beam offset at 2 mm; (**c**) The laser beam offset at 3 mm; (**d**) The laser beam offset at 4 mm.

**Figure 10:**
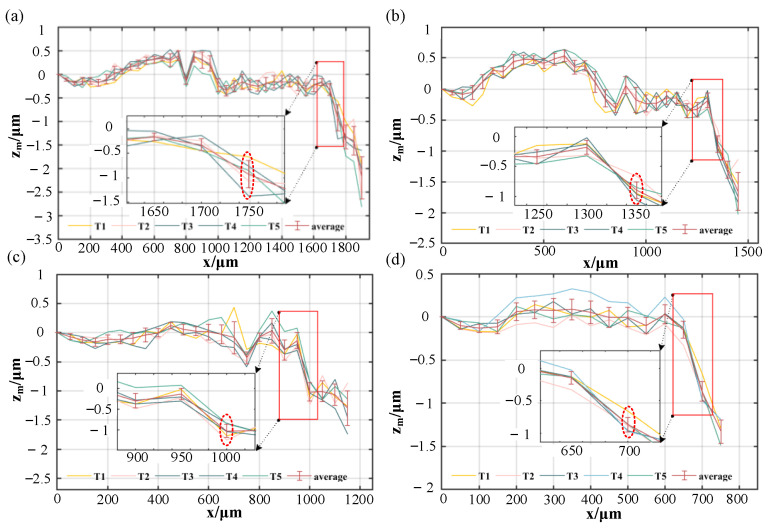
The measurement error when the measured surface tilts clockwise around the y-axis. (**a**) The laser beam offset at 1 mm; (**b**) The laser beam offset at 2 mm; (**c**) The laser beam offset at 3 mm; (**d**) The laser beam offset at 4 mm.

**Figure 11:**
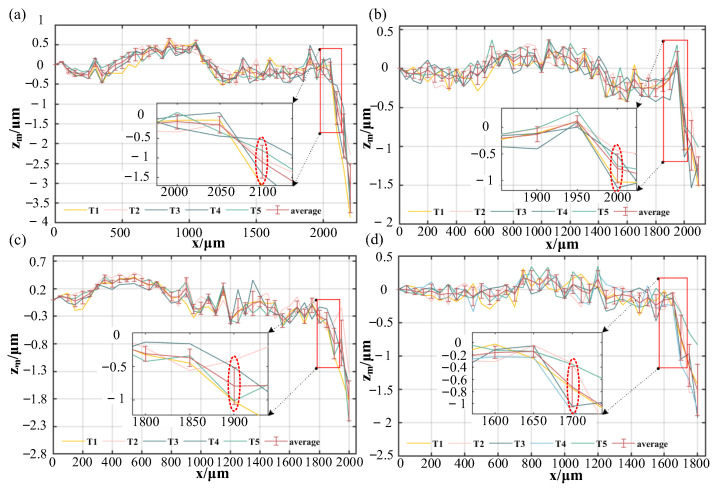
The measurement error when the measured surface tilts clockwise and anti-clockwise around the x-axis. (**a**) The laser beam offset at 1 mm; (**b**) The laser beam offset at 2 mm; (**c**) The laser beam offset at 3 mm; (**d**) The laser beam offset at 4 mm.

Figure 9 shows the measurement error when the measured surface tilts anti-clockwise around the y-axis with the laser beam offset at 1 mm, 2 mm, 3 mm and 4 mm. In Figure 9, Figure 10 and Figure 11, T1, T2, T3, T4 and T5, respectively, represent the experimental data of the five repeated measurements.

The maximum offset distance of the reference sphere within sub-micrometre measurement accuracy is circled with a red ellipse. Applying Formula (5), we can determine the theoretical maximum acceptable tilt angles *A*_1_ for the four offset distances, which are 5.3°, 10.7°, 15.5° and 20.3°. Similarly, Formula (9) allows us to calculate the measured maximum acceptable tilt angles *A*_m1_ with for these four offset distances, yielding values of 5.3°, 10.5°, 15.3° and 20.7°. The analysis in step 1 of Section 2.2 shows that the maximum acceptable tilt angles are not directly affected by the objective lens when the measured surface tilts anti-clockwise around the y-axis, but are determined by the laser incident angle. Under this tilt direction, the maximum acceptable tilt angles can reach 20.3°.

Figure 10 shows the measurement error when the measured surface tilts clockwise around the y-axis for the same offset distances.

Applying Formula (6), we calculate the theoretical maximum acceptable tilt angles *A*_2_ for the four offset distances. The results are 12.3°, 9.7°, 7.1° and 4.7°. Using Formula (9), we determine the measured maximum acceptable tilt angles *A*_m2_ for these offsets as 12.4°, 9.4°, 6.9° and 5.0°. Due to the laser offset, the reflected laser is closer to the tilt direction when the measured surface tilts clockwise around the y-axis. The reflected laser deviates from the objective lens more easily, resulting in a smaller maximum acceptable tilt angle. Under this tilt direction, the maximum acceptable tilt angles can reach 12.3°.

Figure 11 shows the measurement error when the measured surface tilts both clockwise and anti-clockwise around the x-axis for the same offset distances. Furthermore, the laser beam offset remains at 1 mm, 2 mm, 3 mm and 4 mm.

According to Formula (7), the theoretical maximum acceptable tilt angles *A*_3_ with the four offset distances are 14.6°, 13.9°, 13.0° and 11.4°. Using Formula (9), we find the measured maximum acceptable tilt angles *A*_m3_ to be 14.5°, 13.8°, 13.1° and 11.7° for these distances. There is no offset in the projection of the incident laser and reflected laser on the yoz plane, so the maximum acceptable tilt angles are the same when the measured surface tilts clockwise and anti-clockwise around the x-axis. In this tilt direction, the maximum acceptable tilt angles can reach 14.5°.

The experimental findings reveal that the maximum acceptable tilt angle *A*_m1_ increases as the offset distance increases. Conversely, *A*_m2_ and *A*_m3_ decrease with a growing offset distance. Interestingly, there is minimal difference between the theoretical and measured values of the maximum acceptable tilt angles. In practical applications of point autofocus microscopy, the maximum acceptable tilt angles should be the lesser of the theoretical and measured values. Moreover, the surface being measured is recommended to move in the direction of the laser beam offset when it has a large inclination.

## 5. Conclusions

This article constructs a point autofocus microscope, for which a ray-tracing model is established. The functional relationship between the laser beam offset and the maximum acceptable tilt angles with different inclined directions of the measured surface is provided theoretically. To verify the accuracy of theoretical analysis, a novel experimental scheme was proposed. This scheme utilises the geometric features of a precision reference sphere to simulate the deflection angle and displacement of the measured surface. By maintaining the measurement accuracy of point autofocus microscopy within a maximum error of 1 μm, we were able to find the measurement values of the maximum acceptable tilt angles in different directions. The difference between the experiment results and the theoretical results is less than 0.5°. Therefore, the functional relationship formula proposed in this paper can effectively describe the relationship between the maximum acceptable tilt angles and the beam offset accepted when using point autofocus microscopy. This research demonstrates that the laser beam offset affects the maximum acceptable tilt angles differently in various directions for point autofocus microscopy. The microscope’s maximum acceptable tilt angle reaches a peak of 20.3° at a laser beam offset of 4mm, when the tilt direction of the surface measured aligns with the direction of beam offset. Conversely, the microscope’s maximum acceptable tilt angle reaches a maximum value of 12.3° or 14.5° at a laser beam offset 1mm when the tilt direction of the surface measured is opposite or perpendicular to the direction of beam offset. The device demonstrates its strongest ability to measure an inclined surface when measuring in the offset direction of the laser beam.

## Figures and Tables

**Table 1:** The technical parameters of objectives.

Olympus LMPlanFL N 50 × Object Lens		Units
NA	0.5	
Working Distance	10.6	mm
Focal Length	3.6	mm
Resolution	0.67	μm

**Table 2:** The technical parameters of reference sphere.

Precision Balls		Units
Diameter	15.8756	mm
Roundness	0.06	μm

**Table 3:** The technical parameters of objective scanner.

P73.Z200S		Units
Travel	200	μm
Resolution	5.5	nm
Positioning Error	±0.6	μm
Repeatability	±0.5	μm

**Table 4:** The technical parameters of PSD sensor.

PDP90A		Units
Saturation Power	100	μw
Minimum Power	20	μw
Resolution	0.75	μm
Sensor Size	9 × 9	mm

**Table 5:** The technical parameters of high-precision incremental-length gauge.

MT 2500		Units
Measurement Range	25	mm
Position Error	0.2	μm
Repeatability	0.02	μm

## Data Availability

The data presented in this study are available on request from the corresponding author.
